# Human lung carcinomas synthesize immunoregulatory glucocorticoids

**DOI:** 10.1038/s41435-023-00194-y

**Published:** 2023-01-18

**Authors:** Verena M. Merk, Leonie Grob, Achim Fleischmann, Thomas Brunner

**Affiliations:** 1 Chair of Biochemical Pharmacology, Department of Biology, University of Konstanz, Konstanz, Germany; 2 Pathology, Kantonspital Münsterlingen, Münsterlingen, Switzerland

**Keywords:** Immune evasion, Immunosurveillance

## Abstract

The need for new options in lung cancer treatment inevitably leads back to basic research. The tumor itself and the tumor environment especially the interaction with the immune system need to be better understood to develop targeted therapies. In the context of lung cancer glucocorticoids (GC) are mainly known as a combination drug to attenuate side-effects of chemotherapies. However, endogenous extra-adrenal GC have been shown to substantially regulate local immune responses within various tissues, including the lung. In this study we investigated whether primary lung tumors have maintained the capacity to synthesize GC and may thereby regulate anti-tumor immune responses. We show that several non-small cell lung carcinoma (NSCLC) and small cell lung carcinoma (SCLC) cell lines express key steroidogenic enzymes and synthesize bioactive GC under steady state conditions. We also show that tumor-derived GC can inhibit splenic T cell activation, thus demonstrating their immunoregulatory potential. Moreover, steroidogenic enzymes were detected by quantitative RT-PCR and immunohistochemistry in tissue sections of different human lung tumors, further strengthening the idea that human lung carcinomas regulate their microenvironment by releasing immunoregulatory GC, which potentially contributes to immune evasion and treatment resistance.

## Introduction

Lung cancer is one of the most commonly diagnosed cancers worldwide with a poor prognosis depending on the subtype and stage of the cancer [1]. Based on histological characteristics, lung carcinoma can be classified into two main categories, non-small cell lung carcinoma (NSCLC) and small cell lung carcinoma (SCLC), which represents a subtype of neuroendocrine tumors. While early stages of NSCLC can be treated by surgical resection and chemotherapy, diagnosis of SCLC often occurs at advanced stages, and remaining treatment options are limited to chemo- and radiation therapy with a generally low 5-year survival rate [2]. Therefore, intensive research is being conducted on new treatment options, and basic research on lung tumors and the tumor environment may identify novel therapeutic targets.

Glucocorticoids (GC) are steroid hormones with potent immunosuppressive and -modulating activities [3]. They suppress inflammatory processes by interfering with nuclear factor ‘kappa-light-chain-enhancer’ of activated B-cells (NFκB) and activator protein 1 (AP-1) activation, and thus modulate the expression of numerous inflammatory cytokines by immune effector cells. Furthermore, GC induce apoptosis in various immune cells, including T cells and dendritic cells, thereby eliminating key regulatory cells involved in various types of immune responses [4]. The anti-inflammatory effect of synthetic GC has been successfully used for decades in the treatment of inflammatory diseases in all organs of the human body. Moreover, GC are often also co-administered to cancer patients to mitigate the side-effects of chemotherapies [5].

Endogenous GC are primarily produced in the adrenal glands in response to physical, emotional and immunological stress. However, research over the past 20 years revealed that GC are also produced in extra-adrenal tissues, such as the intestine or the skin, and that extra-adrenal GC have a critical role in regulating local immune responses in these tissues [6–9]. Noteworthy, also tumors derived from these tissues are capable of producing immunoregulatory GC and may thus regulate anti-tumor immune responses [10–13]. We have previously shown that the lung tissue produces GC locally under homeostatic and inflammatory conditions [14]. Given that GC receptor (GR) signaling has been associated with cancer cell dormancy and drug tolerance in lung cancer [15], we hypothesized that lung cancer cells may have maintained the capability to produce GC, which may contribute to immune evasion and treatment resistance. In this study we thus analyzed the potential of lung cancers to express steroidogenic enzymes, to produce bioactive GC and to suppress immune cells.

## Results & discussion

**Fig. 1:**
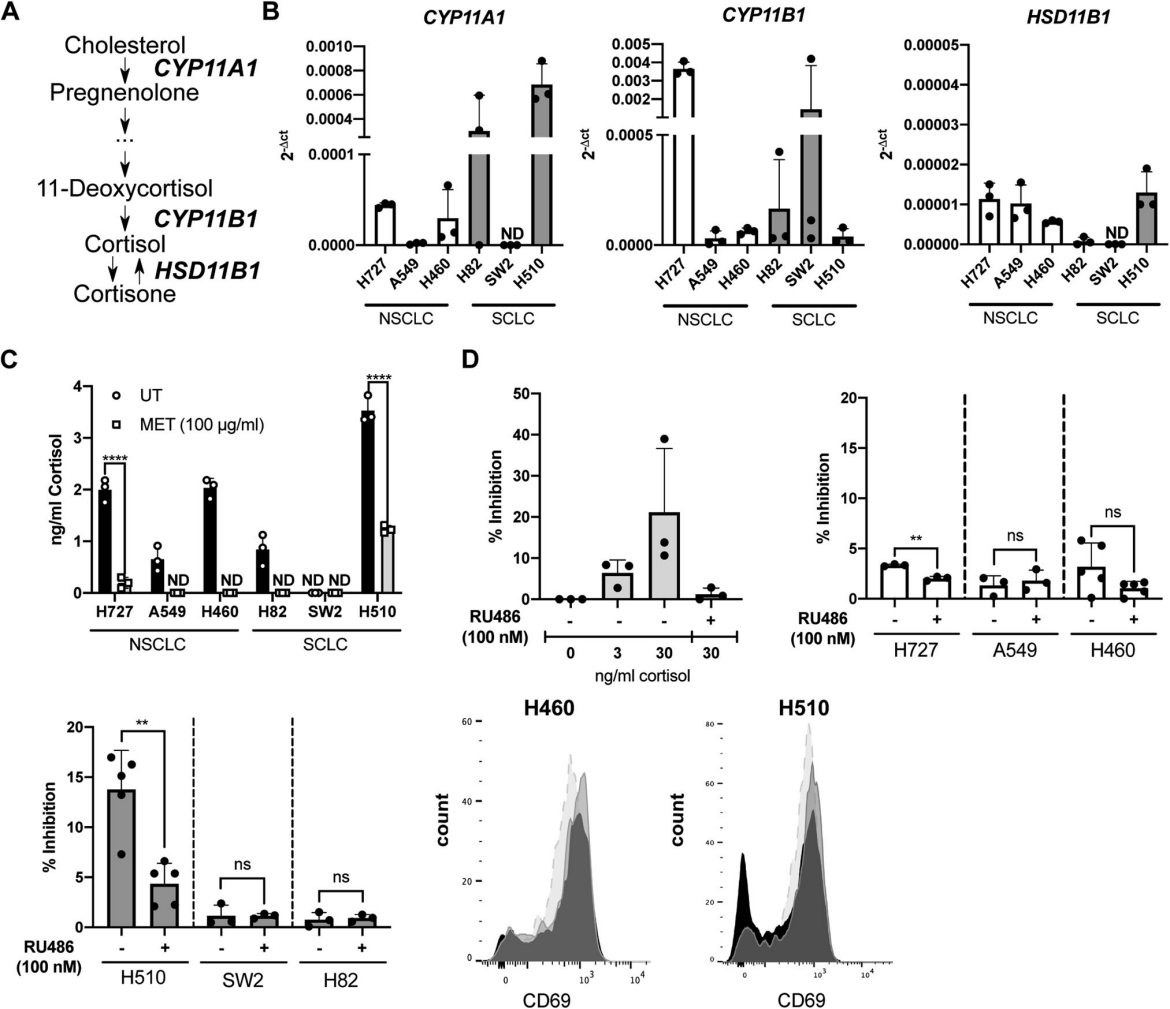
Human lung cancer cell lines produce immunoregulatory glucocorticoids. **A** A simplified scheme of the glucocorticoid (GC) synthesis pathway in humans. **B** Expression of *CYP11A1*, *CYP11B1* and *HSD11B1* was assessed in non-small cell lung cancer (NSCLC) and small cell lung cancer (SCLC) cell lines by RT-qPCR. Expression levels were normalized to *β-ACTIN*. Bars indicate mean values + SD (*n* = 3). **C** GC concentration in cell culture supernatant of control or metyrapone-treated cells (MET) was assessed in the GC bioassay. Bars show mean values + SD (*n* = 3) **D** Primary murine splenocytes were pretreated with buffer control or RU486 (100 nM) for 1 h, prior to treatment with 0, 3 or 30 ng/ml cortisol, or lung cancer cell line supernatant. After 2 h cells were activated with concanavalin A (1 µg/ml) and CD69 expression in CD4 + T cells was analyzed by flow cytometry after 20 h. Mean values + SD of *n* = 3 (cortisol titration) or *n* = 3–5 (cell culture supernatants) experiments are shown. Representative CD69 expression profiles for CD4 + T cells treated with H460 and H510 cell culture SN: dashed light gray plot = activated CD4 + T cells, black plot = treated with cell culture SN, gray plot = treated with cell culture SN plus RU486. Statistical analysis was performed by using Two-way ANOVA multiple comparisons (**C**), or two-tailed unpaired *t*-test (**D**). ** = *p* < 0.005; *** = *p* < 0.0005; **** = *p* < 0.0001; ns not significant, ND not detectable.

In order to investigate the capability of lung cancers to produce GC, we characterized five NSCLC and five SCLC cell lines. Here, we show that various lung cancer cell lines express the genes encoding for P450(scc) (*CYP11A1)*, 11β-hydroxylase (*CYP11B1)* and 11β-hydroxysteroid dehydrogenase 1 (*HSD11B1)*, which are key enzymes of the GC synthesis pathway (Fig. 1A, B). *CYP11A1* and *CYP11B1* are genes encoding crucial enzymes in the de novo synthesis pathway of cortisol from cholesterol, while the *HSD11B1* gene encodes the enzyme responsible for the reactivation of inactive cortisone to cortisol (Fig. 1A). We also confirmed actual synthesis and bioactivity of tumor-derived GC in a luciferase-based bioassay. The cell lines investigated produced different amounts of bioactive GC, which were released into the cell culture supernatant. Addition of the GC synthesis inhibitor metyrapone to the cells results in either a significant inhibition or complete block of GC synthesis in all cell lines tested (Fig. 1C). Metyrapone mainly inhibits the steroidogenic enzymes encoded by the *CYP11A1*, *CYP11B1* and *HSD11B1* genes, and thus both GC synthesis pathways, de novo synthesis and reactivation. The absence of GC in the cell culture supernatant of SW2 cells is consistent with the absence of detectable *CYP11A1* and *HSD11B1* expression. In contrast, the comparably high amount of GC in the supernatant of H510 cells might be related to the high expression of *CYP11A1* (Fig. 1B, C). P450scc catalyzes the first reaction of the GC de novo synthesis pathway (cholesterol to pregnenolone) and is thus rate-limiting for the generation of active GC.

From these findings we concluded that most lung cancer cell lines are capable of producing active GC. We thus aimed next to place this observation in the context of tumor immune evasion. It is well known that GC inhibit T cell activation, which may represent an important tumor immune evasion mechanism. Previously, we demonstrated that colon carcinoma-derived GC inhibited splenic T cell activation, as monitored by CD69 upregulation [10]. For this reason, we repeated this experiment in the presence or absence of lung cancer cell culture supernatant from an NSCLC (H460) and an SCLC (H510) cell line. The principle of this T cell suppression assay was confirmed by the addition of cortisol prior to T cell activation, which resulted in decreased expression of the early T cell activation marker CD69. The inhibition was reversible by adding the GR antagonist RU486, confirming GC specificity (Fig. 1D). A suppression of T cell activation was also detected when splenocytes were exposed to the cell culture supernatant of H510, H727 and H460 cells, but not with cell culture supernatant of A549, SW2 and H82 cells. H510 supernatant appeared to be the most potent compared to that of all other cell lines tested (Fig. 1D). This is consistent with the amounts of GC in the supernatant of the cell lines that were determined in the luciferase-based bioassay (Fig. 1C). The higher the amount of produced GC, the more T cell inhibition was observed.

**Fig. 2:**
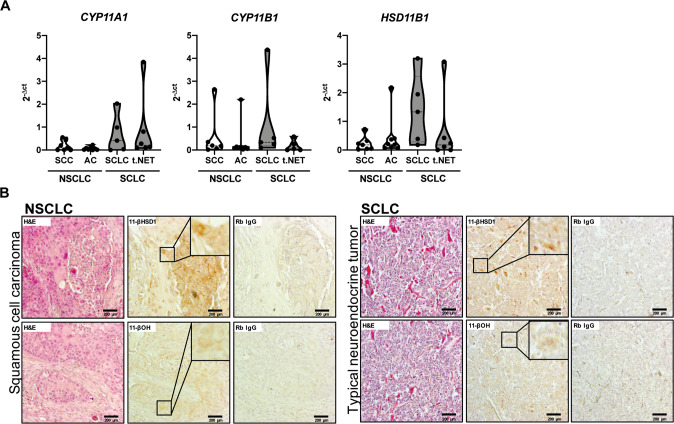
Different types of human lung cancers express steroidogenic enzymes. **A** Expression of *CYP11A1*, *CYP11B*1 and *HSD11B1* was assessed in tissue sections of non-small cell lung carcinoma (NSCLC, SCC = squamous cell carcinoma, AC = adenocarcinoma) and small cell lung carcinomas (SCLC, t.NET = typical neuroendocrine tumors) by RT-qPCR. Violin plots of *n* = 5–8 tumor samples are shown. **B** Immunohistochemistry analysis of 11-β hydroxysteroid dehydrogenase 1 (11-βHSD1) and 11-β hydroxylase (11-βOH) in tissue sections of squamous cell carcinoma (NSCLC) and typical neuroendocrine tumor (SCLC). H&E stainings and Rb IgG isotype controls are shown for comparison. Scale bar = 200 µm. Inlays show magnifications.

These finding suggest that tumor-derived GC could indeed represent a possible immune evasion mechanism in lung cancer patients. Therefore, we aimed to confirm the expression of steroidogenic enzymes in tissue samples from lung cancer patients. These analyses revealed that *CYP11A1*, *CYP11B1* and *HSD11B1* mRNA were expressed in all lung cancer types analyzed, i.e., squamous cell carcinoma (SCC), adenocarcinoma (AC), typical carcinoid/neuroendocrine tumor (t.NET) and SCLC (Fig. 2A). Although not significant, there is a trend toward higher expression levels of steroidogenic enzymes in SCLC. Therefore, it is striking that the SCLC cell line H510 had the highest GC production in vitro. This may indicate a more important role of GC synthesis in this aggressive form of lung cancer. Considering the limited therapeutic options for SCLC, this interesting observation encourages further investigations on tumor GC synthesis as novel therapeutic target. In addition, the steroidogenic enzyme expression in patient samples was further confirmed at the protein level by immunohistochemical detection of 11β-hydroxylase 1 (*CYP11B1*) and 11β-hydroxysteroid dehydrogenase 1 (*HSD11B1*) (Fig. 2B). In conclusion, we show that various lung cancer cell lines are capable of producing bioactive GC that have the potential to inhibit T cell activation. Considering that GC co-therapy has been reported to interfere with chemotherapy by the inhibition of pro-apoptotic signaling and the induction of pro-survival genes [16, 17], our results further indicate that the application of GC to mitigate side-effects of chemotherapy should be reconsidered also in terms of tumor immune evasion. Finally, together with the data from human patients our study provides first evidence that lung tumor-derived GC may modulate their microenvironment and suppress anti-tumor immune responses. It further suggests that interfering with GC synthesis pathways in tumor cells may represent a novel treatment option in lung cancer therapy. Therefore, chemotherapy in combination with pharmacological inhibitors of GC synthesis could be a potential strategy, to induce tumor cell death, while supporting the immune system to fight the tumor.

## Materials & methods

### Cell culture

H727, H460, H82, SW2, and H510 cells cultured in RPMI-1640 (Sigma-Aldrich), containing 10% FCS, 50 µg/ml gentamycin, 2,5 mM L-glutamine, A549 in DMEM (Sigma-Aldrich), containing 10 % FCS, 50 µg/ml gentamycin, 2,5 mM L-glutamine. All cell lines were originally from ATCC and were received from Nycomed. They were tested for mycoplasma contamination prior to experiments. For GC bioassay and T cell activation assay cells were cultured for 24 h in phenol red-free, 5% steroid-free FCS containing DMEM (50 µg/ml gentamycin, 2,5 mM L-glutamine). The supernatant was collected and boiled for 15 min at 90 °C.

### Luciferase-based GC bioassay

GC concentrations in cell culture supernatants were determined via a GR-regulated luciferase reporter assay [14]. HEK293T cells were transfected with a GR expression construct (SVGR1), a GR response element (GRE)-containing luciferase reporter construct (GRE2tk-LUC) and β-galactosidase for normalization of transfection efficiency. Transfected HEK293T cells were treated with cell culture supernatant or cortisol standard, and luciferase activity in cell lysates was assessed after 16 h. GC concentration in cell culture supernatant was calculated from the cortisol standard curve.

### T cell activation assay

Primary murine splenocytes were isolated from C57BL/6 mice and exposed to cell culture supernatant or cortisol standard. In some conditions, the GR was blocked by 1 h pre-treatment with 100 nM of the GR antagonist RU486. After 2 h, splenocytes were activated with 1 µg/ml of the lectin concanavalin A for 20 h. Splenocytes were stained with anti-CD3-BV605 (BioLegend, #100237), anti-CD4-FITC (BioLegend, #100406), anti-CD8-AF700 (BioLegend, #100730), anti-CD69-PE (BioLegend, #104508) and DAPI, and analyzed by flow cytometry on a LSR Fortessa (BD). Single cells were gated for live, CD3+, CD4+ and CD69 + T cells.

### RT-qPCR

Total RNA isolation from cells with RNA-Solv® reagent (OMEGA bio-tek) and total RNA isolation from paraffin-embedded human tissue with RNeasy FFPE Kit (Qiagen) was performed according to the manufacturer’s protocol. High-Capacity cDNA Reverse Transcription Kit (Applied Biosystems, Foster City, USA) was used to reverse transcribe 2 µg of DNAse-treated RNA into complementary DNA (cDNA). RT-qPCR was performed on a QuantStudio3 PCR system using Fast SYBR Green Master Mix (applied biosystems), 0.5 µM forward and reverse primers and a 1:10 dilution of the cDNA. Expression levels were normalized to *β-ACTIN*. The following primers were used:

*hCYP11A1* (fwd AAGGTGTCAGCAGGTTCTGTGTCT; rev TCTCTGTGAGCTGTCTTGCCCTTT)

*hHSD11B1* (fwd: GTTACGTGGTCCTGACTGTAGC; rev: GCAGCAACCATTGGATAAGCCAC)

*hCYP11B1* (fwd: AAGGTGTCAGCAGGTTCTGTGTCT, rev: TCTCTGTGAGCTGTCTTGCCCTTT)

*hβ-ACTIN* (fwd: CATGTACGTTGCTATCCAGGC; rev: CTCCTTAATGTCACGCACGAT)

### Immunohistochemistry

Investigations on human patient samples have been conducted in accordance with the local ethics committees (ethics committees of the Canton Thurgau and Eastern Switzerland) and the Declaration of Helsinki and informed consent was obtained from all subjects. Formalin-fixed and paraffin-embedded lung cancer tissue sections were provided by Achim Fleischmann (Kantonspital Münsterlingen, Switzerland). Heat-induced antigen retrieval was performed in sodium citrate pH 6,0 (anti-CYP11B1 antibody) or Tris-EDTA pH 9,0 (anti-11β-HSD1 antibody) buffer. Endogenous peroxidase was blocked with 1% hydrogen peroxide (Sigma-Aldrich). The sections were stained using a rabbit anti-human 11β-HSD1 antibody (ab39364, Abcam), rabbit anti-human CYP11B1 antibody (HPA056348, Sigma) or rabbit isotype control, and a biotin-labelled secondary antibody. Vectastain ABC-kit (Vector Laboratories, Burlingame, CA, US) was used to convert the substrate DAB (Roche) into a brown colored product. Nuclei were visualized with hematoxylin (Roth).

### Statistical analysis

Statistical analyses were performed using GraphPad Prism (v.8.0; La Jolla, CA, USA). Data were tested for normal distribution with the Shapiro Wilk and the Kolmogorov–Smirnov test and *F* test was performed to compare variances between groups. Details of statistical tests are indicated in the respective figure legend. All statistical analyses were considered as statistically significant at a probability level of *p* < 0.05.

## Data Availability

The datasets generated during and/or analyzed during the current study are available from the corresponding author on reasonable request.
